# Polyhydroxybutyrate Metabolism in *Azospirillum brasilense* and Its Applications, a Review

**DOI:** 10.3390/polym15143027

**Published:** 2023-07-13

**Authors:** María de los Ángeles Martínez Martínez, Lucía Soto Urzúa, Yovani Aguilar Carrillo, Mirian Becerril Ramírez, Luis Javier Martínez Morales

**Affiliations:** Centro de Investigaciones en Ciencias Microbiológicas, Instituto de Ciencias, Benemérita Universidad Autónoma de Puebla, Av. San Claudio y Av. 24 Sur, Col. San Manuel Ciudad Universitaria, Puebla 72570, Mexico; angeles.martinezm@correo.buap.mx (M.d.l.Á.M.M.); lucia.soto@correo.buap.mx (L.S.U.); yovani.aguilarc@alumno.buap.mx (Y.A.C.); mirian.becerrilr@alumno.buap.mx (M.B.R.)

**Keywords:** polyhydroxybutyrate, *Azospirillum brasilense*, PHB genes, PHB regulation, PHB metabolism

## Abstract

Gram-negative *Azospirillum brasilense* accumulates approximately 80% of polyhydroxybutyrate (PHB) as dry cell weight. For this reason, this bacterium has been characterized as one of the main microorganisms that produce PHB. PHB is synthesized inside bacteria by the polymerization of 3-hydroxybutyrate monomers. In this review, we are focusing on the analysis of the PHB production by *A. brasilense* in order to understand the metabolism during PHB accumulation. First, the carbon and nitrogen sources used to improve PHB accumulation are discussed. *A. brasilense* accumulates more PHB when it is grown on a minimal medium containing a high C/N ratio, mainly from malate and ammonia chloride, respectively. The metabolic pathways to accumulate and mobilize PHB in *A. brasilense* are mentioned and compared with those of other microorganisms. Next, we summarize the available information to understand the role of the genes involved in the regulation of PHB metabolism as well as the role of PHB in the physiology of *Azospirillum*. Finally, we made a comparison between the properties of PHB and polypropylene, and we discussed some applications of PHB in biomedical and commercial areas.

## 1. Introduction

Gram-negative *Azospirillum brasilense* belongs to the α-proteobacteria class. It is a motile, vibrio-shaped bacterium of 2.0–4.0 μm length [1]. *Azospirillum* promotes plant growth. Also, it produces high quantities of bioplastic called poly-β-hydroxybutyrate (PHB) [2]. PHB is part of a cluster of bioplastics called polyhydroxyalkanoates (PHA). There are more than 150 different PHAs discovered. The two PHAs most studied are polyhydroxyvalerate (PHV) and PHB [3].

PHB is a biodegradable and biocompatible plastic characterized to have a methyl radical in the β-position of the carbon skeleton of PHA [4]. It has been shown that *A. brasilense* produces only 3-hydroxybutyrate monomers [5,6]. Fourier-transform infrared spectroscopy (FTIR) analyses have shown an ester band v(C=O) at 1727 cm^−1^, which is compatible with PHB [7]. This review aims to summarize the most important factors to consider for understanding PHB metabolism in *A. brasilense*. Throughout the text, we discuss the best carbon and nitrogen sources for improving PHB production. The regulation of PHB metabolism and the functions of PHB are analyzed. Finally, a comparison between the characteristics of PHB and polypropylene (PP) is reviewed, and some examples of uses of PHB in the medical industry are described.

## 2. The Role of the Carbon Source in PHB Production by *A. brasilense*

Previously, it was demonstrated that *A. brasilense* accumulates large quantities of PHB when it grows on a medium supplemented with high concentrations of carbon with minimal quantities of nitrogen (high C/N ratio) [2,8,9]. *Azospirillum* can use a wide range of carbon and nitrogen sources. In terms of carbon, it grows well in fructose, malate, succinate, oxaloacetate, pyruvate, glycerol, lactate, and β-hydroxybutyric acid, among others [1,10,11]. Amino acids are poorly used as carbon, and glucose cannot support the growth of *A. brasilense* [12,13]. N_2_, amino acids, NH_3_, NH_4_, and NO_3_^−^ have been reported as good nitrogen sources for this bacterium [11,14,15]. *Azospirillum* can use a wide spectrum of carbon and nitrogen sources because in this bacterium occur tricarboxylic acid (TCA), glyoxylate, and Entner–Duodoroff cycles, but it lacks Embden–Meyerhof–Parnas and hexose monophosphate pathways [12,13,16].

To improve PHB synthesis, several carbon and nitrogen sources have been evaluated. The highest quantities of PHB were produced when malic acid and ammonia chloride were used as carbon and nitrogen sources, respectively [5,9,17]. When *A. brasilense* grows on malate and ammonia chloride, it accumulates up to 88% of dry cell weight (DCW) as PHB. The carbon source, malate, enters the TCA cycle to produce both primary and secondary metabolites. 

On fructose or lactate, Azospirillum reaches 40 and 50% of PHB as dry cell weight, respectively [5]. Another nitrogen source that allowed high PHB accumulation was sodium nitrate [18]. *A. brasilense* fixes nitrogen when the nitrogen source is depleted. Under nitrogen-fixing conditions, it accumulates from 30 to 75% of PHB as dry cell weight [8,18,19]. Oxygen is also important for PHB synthesis. Data showed that the use of malate and ammonium chloride in addition to high oxygen levels inhibits PHB accumulation by *A. brasilense* [8]. However, low oxygen availability leads *Azospirillum* to accumulate more than 70% of dry cell weight as PHB [5,9]. Previous studies found the highest accumulation of PHB when a 70–140 C/N ratio was used [2,8].

Another *Rhodospirillaceae*, *Rhodospirillum rubrum*, uses acetate for PHB synthesis and prefers anaerobic conditions to improve it [20]. Pseudomonads turn acetate, ethanol, fructose, glucose, gluconate, and glycerol into acetyl-CoA for PHA synthesis. In this bacterium, PHA metabolites are obtained through β-oxidation and de novo fatty acid synthesis pathways [21]. Other carbon sources used by bacteria to produce PHB are methane for *Methylobacterium* strains [22], mannitol for *Bradirhizobium diazoefficiens* [23], and glucose for *R. eutropha*. The latter accumulates up to 90% of PHB (Table 1) [24]. *R. eutropha*, *Azotobacter* spp., *Bacillus*, *Pseudomonas*, and *Azospirillum* spp. are the most studied microorganisms in terms of PHB production [2,24].

## 3. PHB Synthesis and Degradation by *A. brasilense*

**Figure 1:**
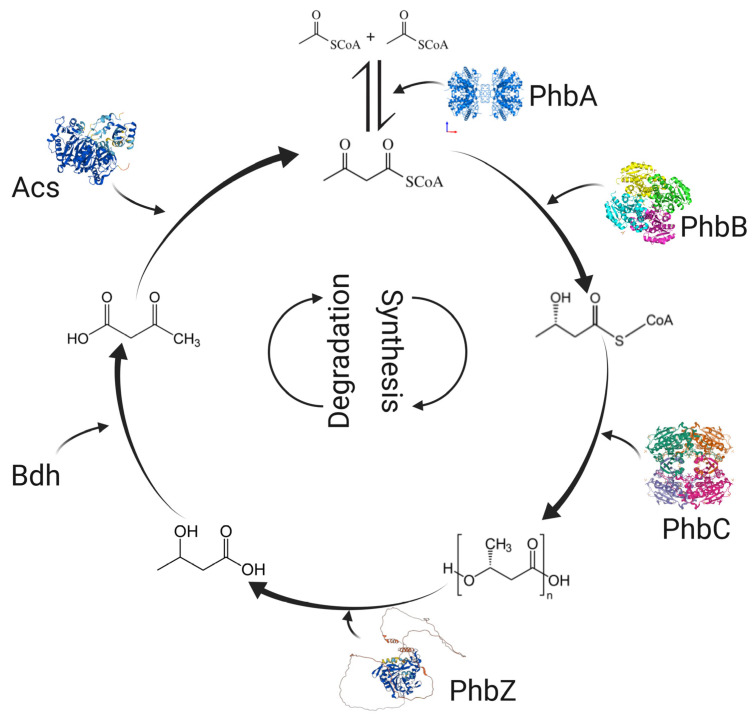
The metabolic pathway for PHB synthesis and degradation. The enzymes involved in PHB synthesis are PhbA (β-ketothiolase), PhbB (Acetoacetyl-CoA reductase), and PhbC (PHB synthase). The enzymes involved in PHB degradation are PhbZ (PHB depolymerase), Bdh (β-hydroxybutyrate dehydrogenase), Acs (Acetyl-CoA synthetase), and PhbA (β-ketothiolase) (Created with data previously reported [32,37,38]).

Biopolymer synthesis by *A. brasilense* involves three enzymatic reactions. The first is catalyzed by β-ketothiolase (coded by the *phb*A gene), which condenses two acetyl-CoA molecules and synthesizes acetoacetyl-CoA. Afterward, it is reduced to β-hydroxybutyryl-CoA by an NAD(P)-dependent acetoacetyl-CoA reductase (coded by the *phb*B gene). Finally, the β-hydroxybutyryl-CoA is polymerized into PHB by the PHB polymerase coded by the *phb*C gene (Figure 1) [32,33]. This pathway occurs similarly in *A. beijerinckii*, *R. eutropha*, and *Sinorhizobium meliloti*, among others [34,35,36]. Other microorganisms such as *P. putida* can synthesize PHB and PHV and copolymers, for example, PHB-*co*-PHV. In PHA synthesis by *P. putida*, the roles of PhaJ, epimerase, and FabG have been described. The PhaJ oxidizes acyl-CoAs into enoyl-CoA. The latter is converted into 3-hydroxyacyl-CoA by an epimerase. Then, 3-hydroxyacyl-CoA is reduced by FabG to form (R)-3-ketoacyl-CoA. Finally, a PhaC polymerizes (R)-3-ketoacyl-CoA into PHA. Another pathway to synthesize PHA in *P. putida* begins with the transacylation of malonyl-CoA and acetyl-CoA with the acyl carrier protein (ACP). The resulting malonyl-ACP and acyl-ACP are condensed into ketoacyl-ACP. Afterward, it is reduced to (R)-3-hydroxyacyl-ACP. Next, PhaG elongates (R)-3-hydroxyacyl-ACP with two carbon units into PHA monomers. To finish, a PhaC polymerizes monomers into PHA [21].

**Figure 2:**
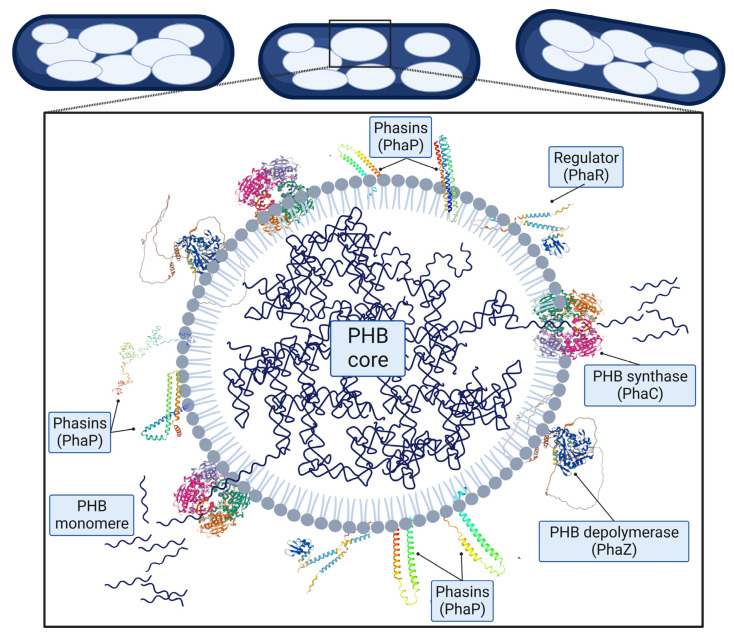
PHB granules in bacteria. Granules contain growing PHB chains at the core and are surrounded by GAPs (Created with data previously reported [39,43,47]).

PHB (and PHA in general) is a hydrophobic material that needs to be stabilized in the cytoplasm. The proteins involved in stabilizing it are known as granule-associated proteins (GAPs). A single PHB granule (carbonosome) contains 98% polymer and 2% GAPs [39,40,41,42]. GAPs include PHB synthases, PHB depolymerases, regulators, and phasins (Figure 2) [43]. PHB synthase and PHB depolymerase initiate PHB synthesis or degradation, respectively [32,38]. Phasins coat and stabilize PHB chains inside bacteria and control the size of the PHB granules [2,43,44,45]. Finally, regulator proteins regulate the expression level of phasins when PHB is synthesized or degraded [46].

PHB degradation occurs when bacteria enter a state of starvation, and the exogenous carbon source is depleted. The resulting products can support bacterial growth, serving as a carbon and energy source [5,38]. PHB mobilization in *A. brasilense* involves a PHB depolymerase (PhaZ) that cuts PHB into β-hydroxybutyrate monomers. Then, an NAD(P)-dependent β-hydroxybutyrate dehydrogenase oxidizes β-hydroxybutyrate monomers to acetoacetate. The subsequent step is to convert acetoacetate into acetoacetyl-CoA by an acetoacetyl-CoA synthetase, and, finally, the acetoacetyl-CoA is hydrolyzed by a β-ketothiolase that releases two acetyl-CoA molecules (Figure 1) [18,37,48]. Acetyl-CoA can enter the TCA cycle, β-oxidation, or glyoxylate pathways, among others, and be used to produce metabolic intermediates and energy to sustain the growth of the bacterium [2,8,29]. *S. meliloti*, *R. eutropha*, and other microorganisms share the same mobilization pathway as *Azospirillum* [36]. In *R. eutropha*, PHB is poorly degraded in the absence of nitrogen [49]. In contrast, nitrogen-fixing bacteria such as *A. brasilense* can mobilize PHB when the nitrogen source is depleted, due to the nitrogenase complex. Previous studies suggest PHB mobilization provides enough energy to sustain nitrogen fixation and two binary fissions [50]. Most PHA-producer microorganisms code for several depolymerase isoenzymes [49]. Since *A. brasilense* can use β-hydroxybutyric acid for growth, this bacterium may have extracellular and intracellular depolymerases. However, more studies are needed to provide us with more information [38].

PHB is accumulated at the middle and the end of the logarithmic growth phases. At the stationary phase, when the carbon source is depleted, PHB begins to be degraded, and it is used to sustain bacterial growth [5,8]. Martínez-Martínez et al. [2] observed that PHB was mainly accumulated after 72 h of growth when a high C/N ratio and microaerophilic conditions were used.

## 4. Studies on PHB Synthesis and Degradation Genes

In *A. brasilense* Sp7, some genes involved in PHB synthesis and degradation have been analyzed. It was shown that a *phb*C mutant strain was unable to accumulate PHB granules after 48 h of growth [32]. On the contrary, a *phb*Z mutant strain accumulated the highest quantities of biopolymer and was incapable of using it [37]. Until now, there are no studies evaluating the effect of deleting the *phb*A gene on PHB synthesis; it may probably be because PhbA has other functions than polymer synthesis. However, a *phb*B mutant strain was reported to be unable to produce PHB. PhbB is the only enzyme implicated in PHB synthesis, and it uses NADH and NAD(P)H as coenzymes [51]. The enzyme β-hydroxybutyrate dehydrogenase has been reported to function as an NAD-dependent tetramer formed by four similar subunits [17]. It was found that NADH, NADPH, pyruvate, and acetyl-CoA inhibit β-hydroxybutyrate dehydrogenase activity [17,37].

**Figure 3:**
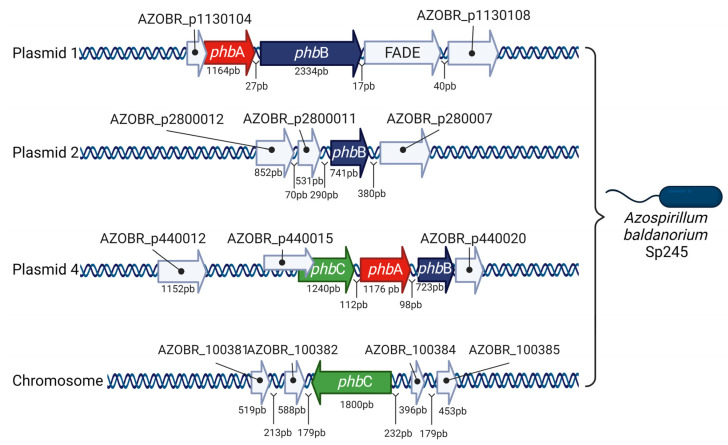
Genes involved in PHB synthesis in *A. baldaniorum* Sp245 (Created with data modified from reference [52]).

Kadouri et al. [32] were the first to report the genetic sequence of genes involved in PHB synthesis by *A. brasilense.* The *pha*A and *pha*B genes were co-transcribed, whereas the *phb*C gene was located in the complementary strand. Recently, the whole available genomic sequence of *A. baldaniorum* Sp245 (formerly named *A. brasilense* Sp245) has shown several copies of PHB genes. In the A. baldaniorum chromosome, a copy of the *phb*C gene was located. The *phb*CAB operon was in plasmid 4. A phbA homolog was found in plasmid 1, whereas copies of phbB were in plasmids 1 and 2 (Figure 3) [52]. In most microorganisms, genes encoding PhbA, PhbB, and PhbC are commonly clustered in an operon [36]. *R. eutropha* and *A. brasilense* contain the *phb*CAB operon, whereas *Azotobacter vinelandii* contains the *phb*BAC operon [36,52,53]. In *P. putida*, the PHA cluster is organized into two operons, *pha*C1ZC2D and *pha*IF [21]. However, there are bacteria with several copies of homologous genes randomly distributed throughout bacterial chromosomes and plasmids [52,54].

Martínez-Martínez et al. [2] analyzed phasin content in the *A. brasilense* Sp7 genome by looking for a phasin_2 domain (PF09361). It was found that this bacterium contains six genes that encode for phasins. The genes were named *pha*P1 (AMK58_RS17065), *pha*P2 (AMK58_RS04265), *pha*P3 (AMK58_RS04270), *pha*P4 (AMK58_RS07520), *pha*P5 (AMK58_RS13850), and *pha*P6 (AMK58_RS20955). Deletion of the *pha*P1 gene showed a phenotype compatible with phasins in other microorganisms. The *pha*P1 mutant strain accumulated fewer PHB granules of a higher size in comparison with the wild-type strain, which accumulated more PHB granules of a lower size.

## 5. PHB Metabolism Regulation in *A. brasilense* Sp7

The PHB pathway is closely related to the TCA cycle because acetyl-CoA molecules are metabolic intermediates. Depending on bacterial needs, acetyl-CoA may be used for one or another [55]. When bacteria are grown on a medium with a high C/N ratio, acetyl-CoA molecules are turned towards the TCA cycle to synthesize the metabolic intermediates required to produce macromolecules such as carbohydrates, proteins, nucleic acids, and lipids. As the TCA cycle is active, high quantities of citrate are synthesized and inhibit the enzymatic activity of citrate synthase. Then, acetyl-CoA molecules are condensed into acetoacetyl-CoA by β-ketothiolase, beginning PHB synthesis [9,54]. The increment of CoA-SH released from acetyl-CoA condensation inhibits β-ketothiolase activity [9]. Excessive acetoacetyl-CoA also inhibits PhbA activity, the key enzyme for PHB biosynthesis [9,56].

Another factor involved in switching on/off PHB synthesis is the redox state of bacteria [39]. Large quantities of NAD(P)H are produced mainly during the TCA cycle and cell respiration. The increase of NAD(P)H is eliminated by acetoacetyl-CoA reductase, which requires NAD(P)H to reduce acetoacetyl-CoA into β-hydroxybutyryl-CoA monomers [39,57]. Then, PHB synthesis leads to an increase in NAD(P) levels. The latter favors the activity of PhaZ that starts the PHB degradation [9,17,21,34,37,39,55,58,59]. *Azotobacter beijerinckii* controls PHB accumulation and mobilization in a similar way as *Azospirillum* [34].

The enzymes β-ketothiolase, acetoacetyl-CoA reductase, PHB synthase, β-hydroxybutyrate dehydrogenase, and acetoacetyl-CoA synthetase are constitutively expressed in *A. brasilense* [9,37]. It seems to be that phasins and regulator proteins are also constitutive [2,60]. For PHB accumulation, *A. brasilense* Sp7 prefers growth in a medium with low oxygen availability. Microaerophilic conditions protect the nitrogenase complex [60]. A high C/N ratio environment allows bacterial growth even if nitrogen is depleted from the medium, due to the nitrogenase complex. Studies by Tal et al. [9] demonstrated that enzymatic activities of β-ketothiolase, acetoacetyl-CoA reductase, and β-hydroxybutyrate dehydrogenase were higher in *Azospirilla* grown in a medium with oxygen limitation.

Although *A. brasilense* Sp7 grows well in ammonia chloride, it has been demonstrated that high ammonia chloride decreases the citrate synthase, isocitrate dehydrogenase, and succinate dehydrogenase activities of *Azospirillum lipoferum*. The effect increases with lower dissolved oxygen (DO). Then, carbon metabolism is restricted to PHB synthesis [61].

## 6. Nitrogen’s Role in PHB Metabolism

*Azospirillum* grows in a medium with a low ammonium concentration. Ammonium assimilation by *Azospirilla* occurs through glutamate dehydrogenase activity that converts glutamate into α-ketoglutarate and ammonia [61]. After ammonium depletion, this bacterium continues growing because it fixes atmospheric nitrogen [62,63]. This process expends a lot of energy, supported by PHB catabolism [50]. The presence of exogenous nitrogen, such as ammonium, nitrate, and nitrite, represses nitrogen fixation [64].

The regulation of nitrogen metabolism in *A. brasilense* Sp7 includes the well-known proteins: GlnD, GlnB, GlnZ, NtrB, and NtrC and other genes involved in the nitrogen fixation process. In the PII-Pz sensing nitrogen system, *gln*B encodes for the PII protein, whereas the Pz protein is coded by *gln*Z. GlnD transfers uridyl groups to GlnB and GlnZ in a medium with nitrogen deficiency [65,66,67]. Under non-nitrogen-limiting conditions, GlnD removes uridyl groups of GlnB and GlnZ. When nitrogen is absent in the growth medium, uridylated GlnB cannot interact with NtrB. Then, NtrB phosphorylates NtrC. NtrC, phosphorylated, induces transcription of genes involved in sensing nitrogen-alternative sources [66,67].

Sun et al. [65] evaluated PHB accumulation by an *A. brasilense* Sp7 *gln*D mutant strain. This mutant was unable to sense nitrogen cell status. Under no nitrogen-limiting conditions, the PII-Pz system in the *gln*D mutant strain was not uridylated/deuridylated. Furthermore, the *gln*D mutant strain accumulated higher amounts of PHB in comparison with the wild-type strain. The *A. brasilense* Sp7 *gln*B–*gln*Z double mutant strain also synthesized higher amounts of PHB than the wild-type strain [65]. In both the *gln*D single mutant strain and the glnB–glnZ double mutant strain, PHB was accumulated during the logarithmic phase of growth.

Sun et al. [58] analyzed the PHB production of *A. brasilense* Sp7 *ntr*B and *ntr*C single mutant strains when ammonium chloride was used as a nitrogen source. The *ntr*B and *ntr*C mutant strains were unable to sense the nitrogen levels when a medium with a low C/N ratio was used for growth, in comparison with the *A. brasilense* Sp7 wild type. As a result, the mutant strains accumulated up to 45% of PHB as dry cell weight, whereas the wild-type strain produced only 10% of PHB under the same conditions. The *ntr*C mutant of *Herbaspirillum seropedicae* accumulated more PHB than the wild-type strain. Also, it was more resistant to oxidative stress [68].

Kukolj et al. [66] analyzed the gene expression profile of *A. brasilense* grown in a medium with low and high nitrogen availability. Bacteria grown under nitrogen-limiting conditions increased the expression of proteins involved in energy production and conversion, signal transduction, and amino acid metabolism. However, when there was no nitrogen limiting, the bacterium increased the expression of proteins related to signal transduction, cell wall biogenesis, coenzyme metabolism, and energy metabolism.

## 7. Flocculation and Cyst Involvement in PHB Production, the Role of Oxygen

Apart from inducing PHB accumulation, media with a high C/N ratio can also stimulate the flocculation of bacteria [11,69,70,71,72]. However, the flocculation phenomenon is present mainly under high oxygen concentrations [11,69,70,73]. For generating energy and fixing nitrogen, the appropriate oxygen concentration for *A. brasilense* is 3–5 μM [62,74]. Under elevated DO *A. brasilense* Sp7 tends to clump. In clumping, motile cells interact between them in response to an elevated aeration rate. Clumped bacteria keep a microaerophilic environment to protect them from excessive oxygen [75]. When high aeration is maintained, clumped cells form irreversible macromolecular aggregates known as floccules, but when high aeration decreases, clumped cells return to a vegetative state [11,55,60,69].

Floccules are macroscopic bacterial aggregates that are clumped by a high accumulation of exopolysaccharides (EPS). EPS form a fibrilar matrix surrounding bacteria. Floccules protect *A. brasilense* Sp7 against the stresses caused by high temperatures (up to 40 °C), desiccation, oxygen availability, and pH [8,11,72,75,76,77,78,79]. *A. brasilense* floccules contain PHB granules with high biopolymer levels [80,81,82,83]. 

The EPS of *A. brasilense* Sp7 contain glucose during the exponential growth phase. However, in the stationary phase growth, they contain mainly arabinose [70,71,84]. EPS, whether glucose-containing or arabinose-rich, served as a carbon source when exogenous carbon was depleted. Interestingly, *A. brasilense* Sp7 cannot grow in media with arabinose or glucose as unique exogenous carbon sources, but it uses its arabinose or glucose as a carbon source [81].

Low nitrogen conditions and a high aeration rate led to *fcl*A overexpression. The *fcl*A gene controls the morphological transition from vegetative to cystic in response to environmental changes. Also, *fcl*A may control nitrogen assimilation by downregulating glutamine synthetase, which synthesizes glutamine from glutamate and ammonia. GlnA (citrate synthase) was also implicated in EPS production. It seems that *fcl*A promotes sugar assimilation by synthesizing carbohydrates for EPS. Also, it was noted that acetyl-CoA was mainly addressed to PHB synthesis rather than the TCA cycle in the *A. brasilense* *fcl*A mutant strain due to the *phb*A gene being overexpressed in an *fcl*A mutant strain [55].

Bible et al. [60] analyzed protein expression during growth under clumping conditions in *A. brasilense* *che*Y1 and the *che*B1–*che*R1 mutants. The *A. brasilense* *che*Y1 mutant strain clumped more than the wild-type strain. On the contrary, the *A. brasilense* *che*B1–*che*R1 double mutant strain was unable to clump. Both *che*Y1 and the *che*B1–*che*R1 mutants showed an increase in the expression of genes involved in PHB metabolism, such as the acetoacetyl-CoA reductase gene and the *pha*P1, *pha*P2, and *pha*P6 genes.

Malinich and Bauer [85] analyzed the transcription profile of encysted *A. brasilense*. Cysts repress genes involved in amino acid biosynthesis, ribosomal biogenesis, and translation. In *A. brasilense* cysts, the *pha*R (AMK58_RS26785) and 3-hydroxyacyl-CoA dehydrogenase (AMK58_19430) genes were also repressed. Genes required for nitrogen metabolism, such as NasT, a response regulator required for growth under nitrate, nitrite/nitrite transporter (AMK58_21400, AMK58_21405, and AMK58_21410), and nitrate reductase (AMK58_21395) were upregulated as well as genes involved in nitrogen fixation [85].

## 8. PHB and Biofilm in *A. brasilense*

Depending on the growth conditions, *A. brasilense* produces a biofilm [86]. Vieruega et al. [87] have reported that the *Azospirillum* biofilm contains proteins such as polar flagellin and OmaA (outer membrane protein), extracellular DNA, and EPS. According to Tugarova et al. [88] and Shelud’ko et al. [89], the *Azospirillum* biofilm also contains high quantities of PHB. PHB would allow bacterial stabilization during biofilm formation.

## 9. Functions of PHB in *Azospirillum brasilense*

PHB accumulation and utilization by bacteria allow its establishment and survival in competitive environments. In such conditions, PHB functions as a carbon and energy source to sustain bacterial growth [18,32,38,84,90]. Also, it was demonstrated that bacteria with high PHB content colonize the rhizosphere to exert beneficial effects on plant growth and crop yield [38,72,78]. The use of inoculants based on *Azospirillum* with a high PHB content can prolong their useful life [38,72,83,90,91,92]. Previous studies have reported that the inability to synthesize or degrade PHB affects the resistance of *A. brasilense* to osmotic and oxidant stresses, as well as its resistance to UV radiation and high temperatures [23]. In *Pseudomonas extremaustralis*, better UV resistance was observed in agreement with a higher PHB accumulation [93]. PHB provides enough energy to sustain growth under nitrogen-fixing conditions. Previous studies have suggested PHB degradation can supply enough energy to sustain nitrogen fixation and spore development [94]. Similarly, PHB synthesis controls the redox state of bacteria, serving as an electron sink [95].

## 10. PHB Properties and Applications 

A comparison between the characteristics of PHB and polypropylene (PP) is listed in Table 2. PHB shares similar properties with PP, in areas such as tensile modulus, tensile strength, and melting temperature. However, PHB is biodegradable and biocompatible, and its degradation does not release toxic products, which does not occur with PP [47]. The above characteristics would make PHB suitable to replace the use of PP in the industry. However, its high production costs, in addition to low thermal stability, a high degree of crystallinity, hydrophobicity, and brittleness, make PHB less suitable for being used in commercial applications [96,97]. To improve the quality of PHB, it has been combined with other materials, which has enhanced its mechanical and physical properties. Some examples of PHB blending are polylactic acid (PLA), hyaluronic acid (HA), polycaprolactone (PCL), polyethylene glycol (PEG), chitosan, and cellulose pectin, among others [97]. Functionalization of PHB by adding epoxy, hydroxyl, carbonyl, phenyl groups, and halogen atoms improves PHB properties [97,98]. It makes it possible to expand the uses of PHB, for example, in drug delivery.

Drug delivery has been one of the most important uses of PHB. Previous studies have shown that PHB can be implanted in the human body, where it causes a mild inflammatory reaction that does not lead to fibrosis or necrosis [101]. Macrophage-mediated inflammation results in an exposition of PHB to extracellular liquids and cells, which results in a slow biopolymer degradation into 3-hydroxybutytyrate monomers and oligomers [102]. These properties suggest PHB is a good candidate for drug delivery [103]. Pandian et al. [104] loaded ursolic acid (an inhibitory agent against the proliferation of tumors) into PHB nanoparticles and evaluated the delivery, availability, and activity of the ursolic acid released from PHB nanoparticles against HeLa cells. In agreement with the number of dead tumor cells, it was concluded that the PHB nanoparticles had released ursolic acid, and they were more effective at 96 h. Another example of drug delivery was reported by Parsian et al. [105], who designed PHB-coated magnetic nanoparticles loaded with gemcitabine (GEM-PHB-MNPs) in order to release gemcitabine for treating breast cancer. Results showed that gemcitabine was released in an acidic microenvironment, like tumors. It was also shown that PHB-MNPs were not cytotoxic to cells. PHB/chitosan blends have been impregnated with ketoprofen [106]. PHB nanospheres and PHB microspheres loaded with extended-spectrum antibiotics have been used to prevent post-surgery infections. Sulbactam ampicillin/cefoperazone, and gentamicin have been loaded into PHB-co-PHV for drug delivery [107]. Other studies on drug release from PHB can be reviewed [97,108,109,110].

PHB can be used for tissue engineering. Deng et al. [111] developed an extracellular matrix of rabbit chondrocytes grown on PHB-co-PHH (polyhybroxybutyrate-co-hydroxyhexanoate) scaffolds. The results showed better seeding on PHB-co-PHH scaffolds than on single PHB scaffolds. It was also shown that more collagen was produced on PHB-co-PHH than on PHB. Temporary stents, bone plates, patches, and screws have been fabricated [111,112]. PHB-based composites were suitable for wound dressing and ocular implants [113]. Also, several artificial tissues, such as retinal, bone, tendon, cartilage, and muscle, have been developed [114,115]. Biopolymers can support cell growth [111,116]. PHB blends can be used as scaffolds and bone implants [97,117].

To be used for packaging, PHB must be stable, flexible, and highly resistant. A good biopolymer must provide a barrier against water vapor, oxygen, and carbon dioxide [118]. PHB blends are promising materials for packaging because they exhibit good barrier properties, and their degradation products are non-toxic for the environment [119]. PHB blends are used in bottles, jars, films, et cetera [120]. Composites of PHB prepared with coconut fibers showed good thermal stability and better tensile properties, making them suitable to be used as plastic bags for recovered seeds and planted for agriculture [121].

## 11. PHB Biodegradability

PHB biodegradability occurs in soils, water, and aerobic and anaerobic environments. It occurs in microorganisms that have extracellular depolymerases. PHB is degraded upon exposure to soil, compost, or marine sediment. It is supposed that there are approximately 10% of PHB-degrading microbes. PHB can be degraded in aerobic and anaerobic environments. In aerobic environments, PHB degradation results in CO_2_ and H_2_O, whereas methane is released in anaerobic environments. PHB degradation depends on microbial activity, moisture, temperature, pH, molecular weight of PHB, et cetera. PHB degradation units can be processed throughout the β-oxidation and TCA cycles [122].

## 12. Future Outlooks

The metabolic abilities of *A. brasilense* are extensive. It can grow in minimal to rich media and in a wide range of carbon and nitrogen sources. *A. brasilense* accumulates up to 80% of its dry cell weight as PHB, a biopolymer characterized as biodegradable and biocompatible. In bacteria, PHB serves as carbon and energy reserves. Also, it provides resistance to several stressful conditions. Understanding PHB metabolism and regulation in *A. brasilense* may help exploit PHB properties to improve quality of life. In this review were summarized the requirements to improve PHB accumulation by *A. brasilense*. Studies have shown this bacterium accumulates more PHB when it is grown at a high C/N ratio, ranging from 70 to 90. Also, the best carbon and nitrogen sources were malate and ammonia chloride, respectively. A microaerophilic environment is important too.

Although there are some studies on the genes involved in PHB metabolism, these genes have not been fully understood. Further studies are needed to identify other genes implicated in PHB synthesis and degradation. Now that the *A. brasilense* genome is available, it will be possible to analyze other genes that may be involved in PHB metabolism. The National Center for Biotechnology Information (NCBI), the Kyoto Encyclopedia of Genes and Genomes (KEGG), and the Clusters of Orthologous Genes (COG) databases showed that *A. brasilense* contains several copies of genes involved in PHB synthesis and degradation. It would be interesting to know when these genes are expressed and the interactions that occur between each gene, as well as the transcription factors involved in regulating genetic expression. Bioinformatics and in vitro analyses will help us improve our understanding.

PHB is a biodegradable and biocompatible plastic with potential utility for medical and environmental purposes. However, recovering PHB from bacterial cultures is highly expensive. Given that *A. brasilense* is one of the most important microorganisms that produce PHB, it is necessary to develop environmentally friendly techniques that allow us to increase the recovery and purity of the polymer. Also, it is important to make more efforts toward functionalizing PHB in order to expand its uses and to decrease the use of polypropylene.

## Figures and Tables

**Table 1:** PHB accumulation by common strains.

Strain	Carbon Source	%PHB/DCW	Reference
*A. brasilense* Sp7	Malate, fructose, pyruvate	70–88%	[2,8,9,18]
*R. eutropha*	Glucose	80–90%	[25]
*R. rubrum*	Acetate		[20]
*Pseudomonas extremaustralis*	Octanoate, fructose, glucose, glycerol	70–80%	[21,26,27]
*Methylocystis hirsuta*	Methanol:ethanol, methane	73–85%	[28]
*Bradyrhizobium diazoefficiens*	Mannitol, glucose, and glycerol	68%	[29]
*A. vinelandii*	Sucrose	85%	[30]
*Bacillus subtilis*	Various sources	60%	[31]
*Rhizobium nepotum*	Pyruvate	62%	[28]

**Table 2:** Mechanical properties of PHB and PP (modified from [47,97,98,99,100]).

Parameter	PP	PHB
Tensile modulus (GPa)	1.95	3–3.5
Tensile strength (Mpa)	31–45	20–40
Elongation at break (%)	50–400	5–10
Crystallinity (%)	42.6–70	50–60
Melting temperature (°C)	160–176	165–180
Glass transition (°C)	−20–−5	2–9
Density (g/cm^2^)	0.905	1.25
UV resistance	Poor	Good
Biodegradability	No	Yes
Biocompatibility	No	Yes

## Data Availability

Not applicable.

## Abbreviations

PHB: poly-β-hydroxybutyrate; PHA: polyhydroxyalkanoates; PHV: polyhydroxyvalerate; FTIR: Fourier-transform infrared spectroscopy; PP: polypropylene; C/N: carbon to nitrogen ratio; N2: nitrogen gas; NH3: ammonia; NH4: ammonium; NO_3_^−^: ammonium nitrate; TCA: tricarboxylic acid; DCW: dry cell weight; NAD(P: nicotinamide adenine dinucleotide (phosphate); ACP: acyl carrier protein; GAP: granule-associated protein; h:hours; DO: dissolved oxygen; PHB-co-PHH: Polyhydroxybutyrate-co-hydroxyhexanoate; NCBI: National Center for Biotechnology Information; KEGG: Kyoto Encyclopedia of Genes and Genomes; COG: Clusters of Orthologous Genes.
